# Ageing, Leisure Time Physical Activity and Health in Europe

**DOI:** 10.3390/healthcare11091247

**Published:** 2023-04-27

**Authors:** Diego Alvarez-Lourido, José Luis Paniza Prados, Antonio Álvarez-Sousa

**Affiliations:** 1 Hospital Universitario Nuestra Señora de Candelaria, 38010 Santa Cruz de Tenerife, Spain; 2 Department of Sociology, Faculty of Political Science and Sociology, University of Granada, 18071 Granada, Spain; 3 Department of Sociology and Communications Sciences, Group of Territorial Studies (GET), Sociology Faculty, University of Coruna, 15071 Coruna, Spain

**Keywords:** ageing, leisure time physical activity, health-driven motivations, socio-economic status, World Health Organization, European Union

## Abstract

The goal of this article is to analyse leisure time physical activity (LTPA) and health-driven motivations to engage in such activity among elderly people in the European Union. We use as a base the recommendations of the World Health Organisation (WHO) and the theory of the correlation between physical activity according to individual factors (age, gender, socio-economic status) and contextual factors (habitat, community infrastructures, the model of the welfare state of the country of residence). Data are taken from Eurobarometer 88.4. The Generalized Structural Equation Model (GSEM) methodology was used, with the STATA program. The results show that 65.3% of EU citizens over the age of 60 engage in some form of LTPA, that 40.4% do so for health reasons, and that only 32.3% engage in LTPA that meets the minimum guidelines set by the WHO. In addition, there are large differences based on individual and contextual characteristics. The following group was found to be those who practice the most: men, with high socio-economic status, belonging to the middle and upper social classes, living in rural areas where there is infrastructure for physical activity, and above all, in the countries of the Nordic model of social welfare.

## 1. Introduction

The rationale of this study is the need to know the number of older people in the European Union who are engaged in leisure time physical activity (LTPA). It is important to look specifically at physical activity (PA) that meets the WHO minimum requirements for a healthy lifestyle. The study delves into the influence of the correlation of PA when analysing both individual factors (IF) and contextual factors (CF). IFs include socio-demographic and socioeconomic status factors (SES). SES is built on education, income and occupation. We also analysed the influence of subjective social class (SSC). We consider it important to include this second variable as well, as we assume that between two people who have the same SES, if they consider themselves to be of different SSC, they will have different behaviours. However, in addition to the influence of IF, it is necessary to consider the influence of CF such as habitat, the infrastructure of the environment where people live and the welfare state of the country where they live.

In addition, it is necessary to conduct a study based on multivariate analysis in order to verify how each of the controlled variables influences the other variables, for example, the influence of the gender variable when controlled for the influence of SES. The results may be different if analysed at the bivariate or multivariate level. These analyses have not previously been conducted for older people in the European Union. In any case, there are studies that analyse partial aspects. Of particular note, is the study by Stalling et al. [1] in Germany, but it refers to a very specific domain and includes only individual variables but not contextual variables. Other studies such as that of Nikitara et al. [2] measure physical activity in 28 European countries, but they are limited to the age range of 18–64 years and do not address the age range of over 60 years that we have analysed. Lubs et al. [3] conducted a study which analyses the problem of IPA (insufficient physical activity) in 27 countries in Europe, but it is limited to the 65–75 age group. In short, the issue is important and unanalysed.

The WHO [4,5,6] defines physical activity as movement during leisure, transport, work or housework. In a similar vein, we can find definitions from different authors (Caspersen et al. [7], Dubbert [8], Rice and Howel [9], Thivel et al. [10] and Stalsberg and Pedersen [11]). We can differentiate between these four domains of PA: transport PA (TPA), occupational PA (OPA), housing PA (HPA) and leisure time PA (LTPA). We analyse LTPA. The WHO also considers that, in order to be effective, such activity must be practised with a certain frequency and intensity: at least three days a week, with a minimum of 75 min per week of vigorous intensity or 150 min of moderate intensity.

In order to find out about Europeans’ PA, the Eurobarometer asks about the physical activity performed, but it is limited to LTPA. This includes sporting exercise (such as swimming, training in a gym or sports club, running in the park, etc.) as well as other activities not done for sporting purposes but rather for leisure (such as cycling from one place to another, dancing, gardening, etc.). The Eurobarometer asks about intensity and frequency, thus enabling our research to analyse the LTPA that complies with the WHO recommendations for older people.

**Figure 1:**
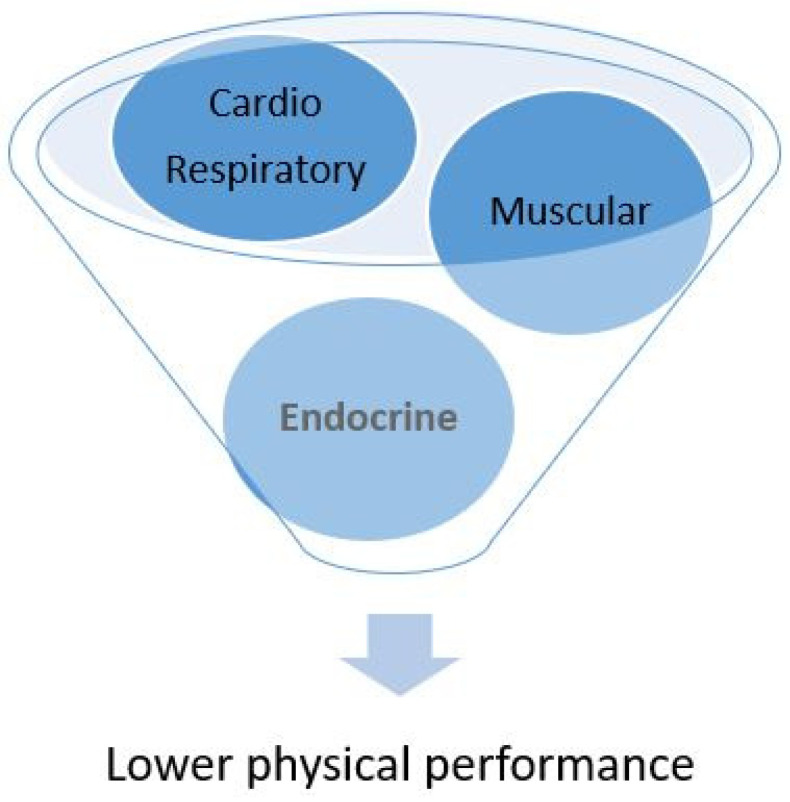
Deconditioning systems. Own preparation.

PA is necessary at all ages, but especially as people grow older, as ageing leads to a reduction in physical performance. This is due to a series of physiological changes involving the neuromuscular, cardiorespiratory and endocrine systems (Figure 1). With ageing, fibrillar atrophy occurs as well as infiltration of the muscle by fat and fibrous tissue. Muscle mass decreases by 2% per year from the age of 50, and muscle strength decreases by 3% per year above the age of 60 [12]. Anaerobic or resistance exercise has been shown to be effective in preventing the loss of muscle mass and strength. At the endocrine level, there is a decrease in growth hormone and sex hormone levels and a state of insulin resistance [13]. Moreover, the functional capacity of the cardiorespiratory system to uptake and distribute oxygen is impaired. Aerobic exercise is the most suitable form of exercise to manage this age-related physical decline.

We start from the idea supported by many researchers [14,15,16,17,18,19,20,21,22,23,24,25,26,27,28,29,30,31,32,33,34] and by the Ministries of Health [35,36,37] in many countries, that socio-demographic factors and socio-economic status, both individual and contextual, are conditioning LTPA. Most studies consider the relationship between LTPA and SES to be positive. Those that think it is negative, rather than measuring LTPA, measure other forms of PA related more to getting to work, physically demanding jobs or housework.

Objective variables (SES) and by subjective self-placement (SSC) are used to measure socio-economic factors at the individual level. At the contextual level, we will take into account the micro (the area where one lives), meso (habitat) and macro (the welfare state model of the country where one lives) contexts [38]. The relationship between SES and PA has been addressed in several articles. More detailed reviews of these articles can be found in Gidlow et al. [39], in Beenackers et al. [40] and in Stalsberg and Pedersen [11]. The two reviews—although different in terms of consideration of the different types of PA, the operationalisation of the concept of SES and the scope of the study in different countries and regions of the world—complement each other to give us an idea of the object of study and its relationship with SES.

The results of previous analyses indicate that when we analyse the LTPA dimension alone, most of the studies show a significant positive association with SES, measured either by a composite indicator (mainly by the variables of occupation, income and education) or by specific component variables, with education being the most important variable in some studies. However, if other PA domains—such as housework, work or active transport—are included, then this association either does not exist or changes signs and becomes negative. Figure 1 of the article by Stalberg and Petersen (2018) clearly concludes that when different types of PA domains are analysed, there is a difference in the LTPA with the other domains (TPA, OPA, HPA). The SES association is positive for LTPA in most of the investigations they analyse (in 22 out of 28 of them) and is predominantly negative, mixed or with no association for the other types of PA.

There are many studies on LTPA and SES, and we are unable to provide a detailed analysis of each of them. Of particular note are the analyses of Kheifets et al. [41], for whom “Among older individuals, multiple SES measures were positively associated with LTPA” (2022, 1). Chen et al. [38] conclude that in their analyses both individual SES and micro- and macro-level contextual factors are associated with LTPA. Marques et al. [28] study the behaviour of Portuguese people and conclude that the higher the socio-economic status, the more positive health-promoting behaviour occurs, either due to “greater motivation or greater access to resources”. Azagba and Sharaf [16] study LTPA in Canada among the population aged 50–79 and find that men with higher education and greater life satisfaction are the least likely to be physically inactive. Lobaszewski et al. [27] study the relationship between LTPA and SES in Poland and conclude that high education and income are associated with high LTPA levels. Borodulin et al. [42] study the relationship between LTPA and education in the population in Finland and find that, as the level of education rises, so does engagement in LTPA. Cerin and Leslie [22] find positive association between income and education in Canada. Azevedo et al. [17] found a strong association between LTPA and socio-economic status in general and education specifically. Satariano et al. [32] also find that education and income are positively and significantly associated with LTPA. One study that does not find positive associations between SES and LTPA is that of Stalling et al. [1], who find a limited relationship, but this is because it considers all PA and not only LTPA.

Among other possible reasons, inequality of PA is likely to be influenced by infrastructural factors; e.g., the area where low-SES people live has fewer infrastructural possibilities than in areas where higher-SES people live, as well as fewer economic resources [23]. However, we consider that this may also be due to motivational aspects; people of lower SES were brought up in a culture where PA was not given importance because they already had physically demanding jobs that consumed their energy, and after work they sought physical rest [11,43,44]. Having been socialised in this culture, even if they have free time, they do not feel motivated to practice PA, which often require organisation and knowledge to engage in them.

Gidlow et al. [39] provide an excellent reflection on the indicators to be taken into account to measure SES, applied to health and PA, analysing the different indicators to be included in the SES: occupation, income and education.

In addition to SES, we also decided to include a subjective indicator, namely subjective social class (SSC), which some authors have found to be a better predictor of health-related issues than objective indicators. Singh-Manoux et al. [45] conclude that even if objective SES is measured, it is essential to include SSC when analysing the factors influencing health. Similar conclusions were also reached by other researchers, considering that “compared with objective indicators, subjective social status was more consistently and strongly related… Most associations remained significant even after controlling for objective social status and negative affectivity” (p. 586) [46]. Frerichs et al. [47] also find significant associations between SSC and PA. Yang et al., as well, find that “there is a positive correlation between physical activity and subjective class identity” (p. 1).

Therefore, we will first test the effect of the objective SES indicators, and then in a multivariate analysis we will add the effect of the SSC. If after analysing the influence of SES we add the SSC, if it shows a significant association, and if it is also found that the model fits better, it means that the SSC has an effect that is added to that of the objective factors and that it is therefore necessary to take SSC into account. Between two people with similar SES, if one of them considers themselves to be of higher social class than the other, the one who considers themselves to be of higher social class is more likely to perform LTPA than the one who considers themselves to be of lower social class. This is because, as Diemer et al. say [48], subjective social class assessments are “not necessarily about determining the accuracy of one’s actual economic position but instead reflect one’s perceived social standing in a community or group in a given sociohistorical context” (p. 105). Although SES is important, it “omits important aspects of social class standing—that is, a person’s perceptions of their relative social standing in relation to others in society” (p. 104). It is for this reason that we decided to include subjective social class (SSC).

On the differences in PA among older people according to socio-demographic differences in age, gender and residence, the review by Sun et al. is noteworthy. *Age* has an influence on PA as well as on health motivation. Throughout the course of life, health situations and practices vary. Booth et al. [49] concluded that from a certain age onward, PA and sporting activity decreases due to factors such as injury or ill health. From youth onward, sport and physical activity decline until the age of 60. As people enter senior adulthood (which we can consider to be over 60 years of age), they become more concerned with health-motivated PA and sport. However, the ability to engage in sport and physical activities with minimum effort declines as the years go by, and this leads to a decrease in practice [50]. Different studies consider that there is a decline in physical activity with age, but some studies consider the decline to be progressive [14,51,52] whereas other studies conclude that there is a sharp decline beginning at the age of 75 [53,54].

Gender influences engagement in LTPA and health-driven motivation [55]. Traditionally, physical activity and, in particular, sport have been more associated with men than with women, which is influencing the inequality of practice as people get older. Perhaps in the future a new culture will change this situation, but gender is currently having an impact. As Alvariñas et al. [56] conclude that “traditionally, the world of physical exercise and sport has been associated with men” (pp. 119–120). In general, studies find that men engage in more LTPA than women [57]. The explanation is sought by other authors in the association between gender and SES, with women of low SES being less likely to engage in LTPA due to a higher priority placed on watching TV or having work commitments [58]. Multivariate analysis is therefore essential for analysing the influence of gender. Dumas and Laberge [50] have found that women from upper-class backgrounds place a hight value on sport and LTPA that is above more immediate material needs and above work, and they even value well-being and health as a goal in itself. When economic capital was secure, they no longer worried about basic needs. This allowed them to engage in daily LTPA of various kinds for aesthetic and health reasons. However, lower-class women are more concerned with achieving economic stability, urgent day-to-day matters, family stability, maternal obligations and other interests that shape their lifestyles, reducing interest in LTPA as its benefits are perceived as more remote. Thus, the effect of gender on LTPA and health-driven motivation is not due to the fact of being male or female butis due to the social conditions associated with gender.

In addition to these factors, other contextual factors play a role. In terms of habitat, we consider that it does have an influence, and there are two different stances on the matter: those who believe that the most active areas are rural areas [59] and those who believe that the most active areas are cities [60]. The existence of infrastructures facilitates the practice of LTPA. When living in a community where adequate infrastructure is in place, LTPA is more likely to occur [61]. The welfare state model of the country in which people live is also significant. After a cross-cultural comparative analysis of sport and welfare state policies, Heinemann concluded that “although the sport systems in these different countries is not a mirror image of the present welfare system, there are clear interconnections’’ [62] (p. 185). Although there are many typologies of welfare states [63], for our study, we decided to use the Esping-Andersen [64] model and its complements for Southern Europe, which are divided into the Nordic model (Denmark, Norway, Sweden, Iceland, Finland), the Anglo-Saxon model (United Kingdom, Ireland), the Continental model (Germany, Austria, France, Belgium, Netherlands, Luxembourg) and the Mediterranean model (Spain, Greece, Italy, Portugal, Malta, Cyprus).

However, the situation is more difficult for the former Soviet Union states that have joined the European Union more recently. We find this model interesting, which divides them on the basis of geopolitical criteria [65], differentiating between those further east in Europe and those further in the centre. While we consider this model to be positive for other matters, in our case, a model that differentiates between those states that are bordering the Nordic model (Estonia, Latvia, Lithuania), those that are bordering the Continental model (Poland, Czech Republic, Slovakia, Hungary, Slovenia) and those that are bordering the Mediterranean model (Croatia, Romania, Bulgaria) seems to us to be more relevant.

Although sport, for some Marxist thinkers, was seen as a typical activity of capitalism [66], in the Soviet Union sport was given great importance [67]. This causes us to think that there is a sporting culture in the states that were part of the Soviet Union that can surpass that of many states of the capitalist system which were not part of the dominant states and that did not have a developed sporting culture and practice, as is the case of the states of the Mediterranean model.

Taking these factors into account, in this investigation, we set out two general objectives which are in turn divided into four specific objectives. The first general objective is descriptive and aims to ascertain the LTPA of the population of the European Union and their health motivations for such activity. This general objective is divided into the following specific descriptive objectives:

- To find out the percentage of the EU population that engages in LTPA considered minimum by WHO.
- To identify the population that performs LTPA for health reasons.

The second general objective refers to the analysis of the explanatory factors that are influencing LTPA and health motivations. This objective is further subdivided into the following specific objectives:

- To study the social conditions at the individual level that influence LTPA and health motivations, which are linked to socio-demographic variables (gender and age), SES and SSC.
- To analyse contextual factors, specifically habitat, the existence of infrastructures for LTPA and the model of the welfare state of the country where people live.

We formulated the following hypotheses to understand the differences in the LTPA and the health motivations of older people in the EU:

**Hypothesis H1.**  *Age is conditioning the population’s LTPA and health motivation. From the 66–70 age group onward, the practice of physical activity starts to decline, as well as the health-driven motivation to practice. The sharp decline occurs from the age of 76 onwards.*

**Hypothesis H2.**  *Gender is conditioning LTPA and health-driven motivation if we measure it at the bivariate level, but if we measure it at the multivariate level and introduce the SES variable, gender will no longer have a share in the influence.*

**Hypothesis H3.**  *As the level of education rises, the more likely people are to engage in LTPA and to have health motivations.*

**Hypothesis H4.**  *As we move up in SSC, people are more likely to engage in LTPA and to have health-driven motivations to be active.*

**Hypothesis H5.**  *Habitat conditions people’s engagement in LTPA. People are more likely to be physically active and motivated to take care of their health in small- and medium-sized cities than in villages and towns and in large cities.*

**Hypothesis H6.**  *Recognising the existence of infrastructures that facilitate the practice of LTPA plays a role in practice and motivation.*

**Hypothesis H7.**  *It is expected that the practice of LTPA and health-driven motivation to do so are higher in the Nordic model, followed by the Continental and Anglo-Saxon models. These are followed in importance by the central and northern EU states that were part of the Soviet Union and finally by the Mediterranean model.*

## 2. Methodology

The methodology used was quantitative, using secondary data from the European Commission, specifically Eurobarometer 88.4 [68], published in 2018, but using data collected in 2017. All 28 countries of the European Union were included. The probability sampling size amounts to 9903 cases of people over 60 years of age. The questionnaires were conducted face-to-face in respondents’ homes.

We analysed three dependent variables: LTPA, intensity of physical activity and health motivations. To measure LTPA, we follow the coding indicated in Supplementary Materials (Table S1). In addition to knowing whether or not individuals engage in LTPA, it is necessary to take into account the intensity with which they do it. WHO distinguishes between vigorous and moderate physical activity, as well as a combination of both.

We performed a cluster analysis in which there are 4 types of people categorized according to LTPA: those who exercise with vigorous intensity, moderate intensity and the combination of both and those who do not engage in the minimum recommended physical activity (see Table S2, in Supplementary Materials).

The independent variables taken into account in the analysis are individual and contextual. The individual variables are socio-demographic and socio-economic. Socio-demographic variables included age and gender. Socio-economic factors include SES and SSC. Cultural includes the level of education. The contextual variables we took into account are the type of community where people live, the opportunities in the area where they live to practice sport and the welfare state model of the country where they live. The independent variables (indicated by acronym) and the categories (indicating the base category for the multivariate analysis) can be found in Table S3 in Supplementary Materials.

Socio-economic status (SES) is a variable that we constructed from three dimensions: educational level, income and occupation. There is no unanimous international agreement among researchers on the dimensions to be taken into account to comprise socio-economic status, but the three indicated are the most widely accepted for analysing its influence on health and LTPA. There is also no agreement on specific indicators for calculating education level, income and occupation. Gidlow et al. [39] establish a discussion to present the analysis, indicating different possibilities used by different authors. After some reflection, in this article we have decided to differentiate between basic education or less (education completed at age 15 or no full-time education), secondary (education completed between ages 16 and 19) and higher education (education completed at age 20 or older). Income is divided into five categories: lower quintile, 2nd quintile, 3rd quintile, 4th quintile and upper quintile. In terms of occupation, we have to differentiate between individuals who are still working and those who are retired. For those who are working, we took the data from the question regarding current occupation. For those who are retired, we took the data from the question regarding their most recent occupation. We classified these occupations according to the typology of Goldthorpe [69,70], with the recommendations of the SEE Working Group to apply to the health sciences (1995), according to which 8 categories of professions can be differentiated. We ultimately established 5, which are as follows: company managers and professions associated with graduate and post-graduate university degrees; professions associated with undergraduate degrees; administrative professionals, personal services, offices, manual work supervisors and salespersons; skilled manual workers and self-employed workers, farmers, stockbreeders, fishermen; unskilled manual workers and housekeepers. With these three dimensions, we constructed the SES index. Although education, income and occupation are usually included, sometimes when operationalised, one of the indicators prevails over the others. Gidlow et al. [39] considered education to be more utilised, and therefore we place slightly more value on it than on the other indicators. Income and occupation assume values of 1, 2, 3, 4 and 5. Education assumes the values of 2, 4 and 6. Thus, the maximum value is 16. Based on statistical criteria, the SES was divided into three groups: lower (up to 6), middle (7–10) and upper (11–16).

The subjective social class (SSC) is based on a Eurobarometer question: “Do you see yourself and your household belonging to…?” The answers are the working class of society, the lower middle class of society, the middle class of society, the upper middle class of society and the upper class of society. For operational purposes, we divide these into three categories: low (the working class of society, the lower middle class of society), middle and upper (the upper middle class of society, the upper class of society).

For the data analysis, we first described the dependent variables. We then performed a bivariate analysis of each of the dependent variables with all the independent variables. Relationships between variables with a Chi-square of less than 0.05 were considered significant. Finally, a multivariate analysis was carried out with the variables that were found to be significant using the GSEM, which was the model required as the variables are categorical. The software Stata (StataCorp LLC, Texas, USA) was used. Initially, all variables that were significant at the bivariate level were included, after which those that were not significant were eliminated. The adjustment of the model was checked to decide on some variables that were significant at the 0.05 level but not at the 0.000 level. After checking the settings, it was decided whether or not to include them. Adjustment was measured using the Akaike Information Criterion (AIC) and the Bayesian (or Schwarz) Information Criterion (BIC).

## 3. Analysis of Results

We will begin by presenting the results by referring descriptively to the evolution of the ageing population in the EU, the LTPA of the population over 60 years of age, LTPA that meets minimum WHO recommendations and their health-driven motivation (dependent variables). Subsequently, we will show a bivariate analysis prior to the multivariable analysis using GSEM.

### 3.1. Descriptive Analysis

The population is ageing at a very fast pace in the EU as a whole and in all countries in particular as shown in Table S4 (Supplementary Materials). Between 2010 and 2021, on average in the 27 countries of the EU, the population aged 65 and over increased from 17.6% to 20.8%, a growth of 3.2%. The country where the population aged the most is Finland, which went from 17% to 22.7%, an increase of 5.7%. The next fastest growing countries in terms of ageing population are Poland (5.1%), Czechia (4.9%), Slovakia (4.7%), etc.; the change in the percentage of ageing population goes downward to reach 0.6% in Luxembourg, but in most countries, it exceeds 3%.

This brings with it a series of social and personal problems that need to be addressed. In addition to the problem of ageing, there is the lack of PA in the general population, which is decreasing significantly due to an increasingly sedentary lifestyle. If we compare the 2013 and 2017 Eurobarometer data for the population aged 15 and over across the EU, we can clearly see a significant increase in the population that never engages in LTPA. Looking at the total population of the EU, the inactivity rate rose from 24.7% in 2013 to 28.7%. In other words, in four years, there was an increase of 4% of the population that does not engage in any LTPA.

The trend was one of increasing inactivity in almost all countries except Cyprus, Bulgaria, Belgium, Greece and Northern Ireland. In the rest of the countries, there was an increase of 1.2% in the Netherlands, 1.4% in Sweden, 2.1% in Finland, 2.2% in Germany East and 2.2% in Malta, Portugal, Spain, Italy, France, Denmark, Luxembourg, Poland, Ireland, Estonia, Slovenia, Latvia, Germany-West, Lithuania, the UK, Hungary and Czech Republic. Particularly of note are Croatia, with an increase of 19.5%, Romania with an increase of 13.4%, Austria with an increase of 10.7% and Slovakia with an increase of 8.6%.

In the EU, 65.3% of its citizens over 60 years of age engage in some form of LTPA (Table S7, in Supplementary Materials). Among those who practice this activity, 40.4% do so for health reasons. However, the population over 60 years of age who practice LTPA and who meet some of the physical exercise guidelines recommended by WHO on average in all EU countries amounts to 32.3%. A total of 67.7% do not meet either of the two guidelines, either because they do not engage in LTPA or because the LTPA does not meet either of the minimum WHO guidelines for intensity (moderate or vigorous) for a healthy lifestyle (Supplementary Materials, Table S5). 

### 3.2. Bivariate Analysis

At the bivariate level, all of the independent variables (age, gender, SES, SSC, type of community in which people live, opportunities for physical activities in the area where they live, welfare state model) show significant associations with the dependent variables we analysed: practice of LTPA, practice of LTPA that meets the minimum required by the WHO and health-driven motivation to practice LTPA (Supplementary Materials, Table S6).

Age is significantly associated with the practice of LTPA, with the likelihood of practising them decreasing as age increases. It starts at 71.2% in the 61–65 age group and ends at 40.2% in the 86 and over age group. The large decline occurs from the 76–80 age group onwards. The same is true for LTPA recommended by WHO. This starts at 38.8% in the 61–65 age group and ends at 9.8% in the 86 and over age group. The large decline also starts in the 76–80 age group. Health-driven motivation for LTPA is also influenced by age, starting at 43.9% in the 61–65 age group and ending at 22.8% in the 86 and over age group.

Gender is significantly associated with LTPA, with women being less likely to engage in LTPA than men; the difference stands at 6.4% (62.4 vs. 68.8). The same is true for LTPA at the intensity recommended by WHO: only 28.7% of women engage in LTPA compared to 36.6% of men. The difference is almost 8%.

The SES significantly influences engagement in LTPA, from 44.9% in the low SES to 65.4% in the medium SES and 84.9% in the upper SES. SES also conditions engagement in LTPA that meets the minimum requirements indicated by the WHO: 17.9% in lower SES, 31.5% in middle SES and 47.3% in upper SES. Health motivations to practice LTPA are also conditioned by SES: 24.9% in lower SES, 39.1 in middle SES and 58.2 in upper SES.

SSC is strongly related to practice LTPA, with an increase in practice as we move up the SSC from lower class to middle class and upper class. When we analyse the performance of LTPA that meets the minimum guidelines required by WHO, there is also an increase as we move up the SSC. The same phenomenon occurs in regard to health-driven motivation to practice LTPA, starting with 30.5% in the lower class, 48.9% in the middle class and 56.4% in the upper class.

The size of the city where people live has significant impact on LTPA and health-driven motivation. People who engage in more LTPA are those who live in small- or medium-sized cities (67.5%), followed by those who live in rural areas or villages (66.2%) and finally those who live in big cities (60.3%). However, in the case of LTPA that complies with WHO recommendations, the highest percentage is found in rural areas or villages (34.4%), followed by small- or medium-sized cities (33.4%) and finally large cities (27.8%).

When the area of residence offers many opportunities for LTPA, the likelihood of doing them is higher (72.3%) compared to areas where there are not many opportunities (48.7%). In addition, there is much higher compliance with WHO recommendations: 37.4% when there are many opportunities and 20.1% when there are not many opportunities. The existence of opportunities is also associated with health motivation for LTPA, with 47.3% responding positively when there are many opportunities and 24.1% responding positively when there are not many opportunities.

The welfare state model of the country in which people live (Table S7) has a key influence on their engagement in LTPA in general, in LTPA that meets WHO recommendations and in health-driven motivation. The countries of the Nordic model lead the way, with no major differences between them. They are followed in importance by the Continental model countries, in the indicators of general LTPA, in LTPA that meets WHO recommendations and in health-driven motivation. Lastly, we have the Mediterranean model countries and the countries of the former Soviet Union bordering them.

### 3.3. Multivariate Analysis with GSEM

The AIC and BIC values were measured to fit the models. For the model of the dependent variable of engaging in LTPA (ltpa) and for the dependent variable of meeting any of the WHO recommended guidelines (ltpawho), the result was that if any of the variables are removed from the model, the adjustment decreases. For this reason, all were included. In the analysis of the dependent variable of health-based motivation to practice sport, the gender variable was not significant, and in that of habitat, although it was significant at the 0.05 level, it was not as significant as other variables at the 0.000 level. The AIC and BIC decrease when taking out the habitat variable, indicating that it associates better and should therefore be removed from the model.

We also examine what happens if we remove the SSC variable from the model, and the result gives us a higher value of AIC and BIC. For this reason, we retain this variable, which is not to say that even after discounting the effect of SES, SSC still has a significant weight in explaining LTPA in general, LTPA that meets WHO recommendations and health-driven motivation for LTPA.

**Figure 2:**
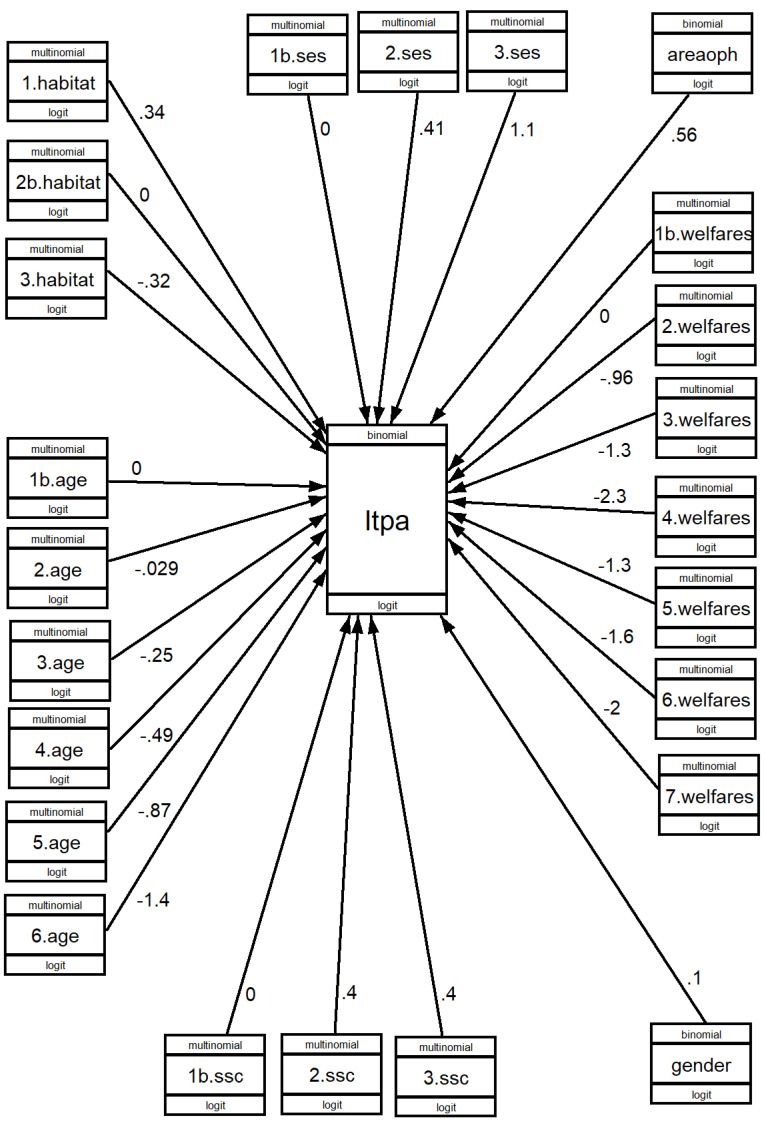
GSEM analysis: engaging LTPA according to social conditions. B values.

**Figure 3:**
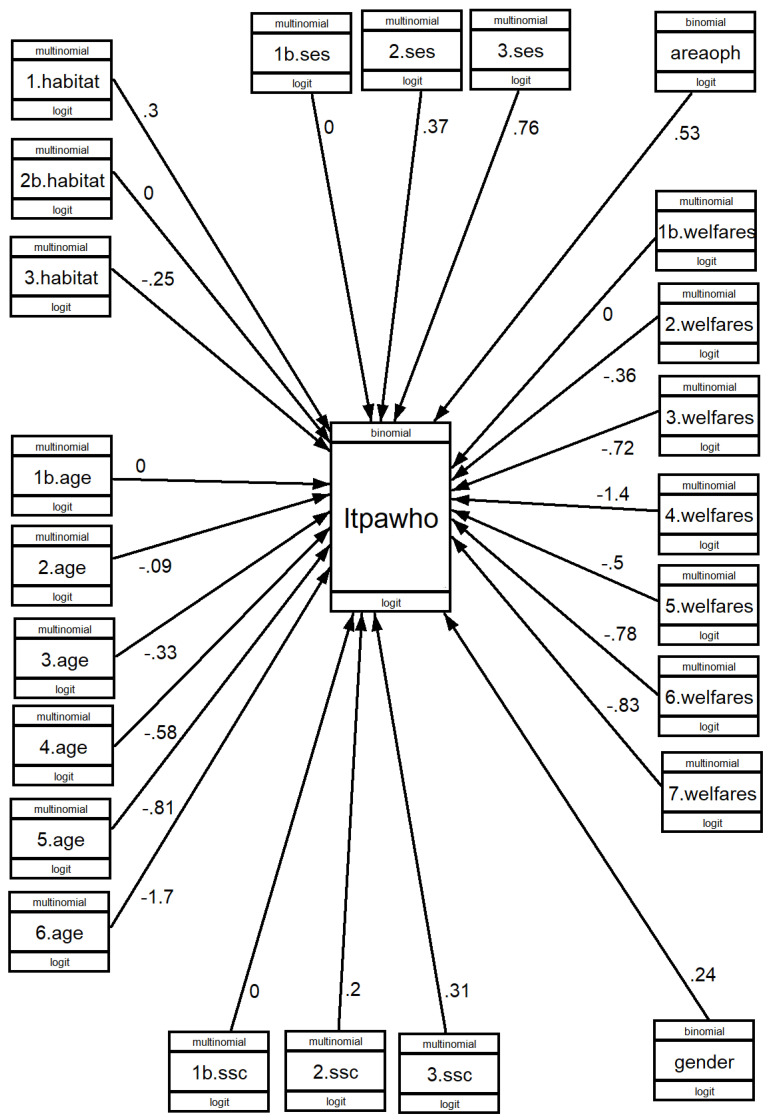
GSEM analysis of engaging in LTPA that meets the minimum WHO recommendations, according to social conditions. B values.

**Figure 4:**
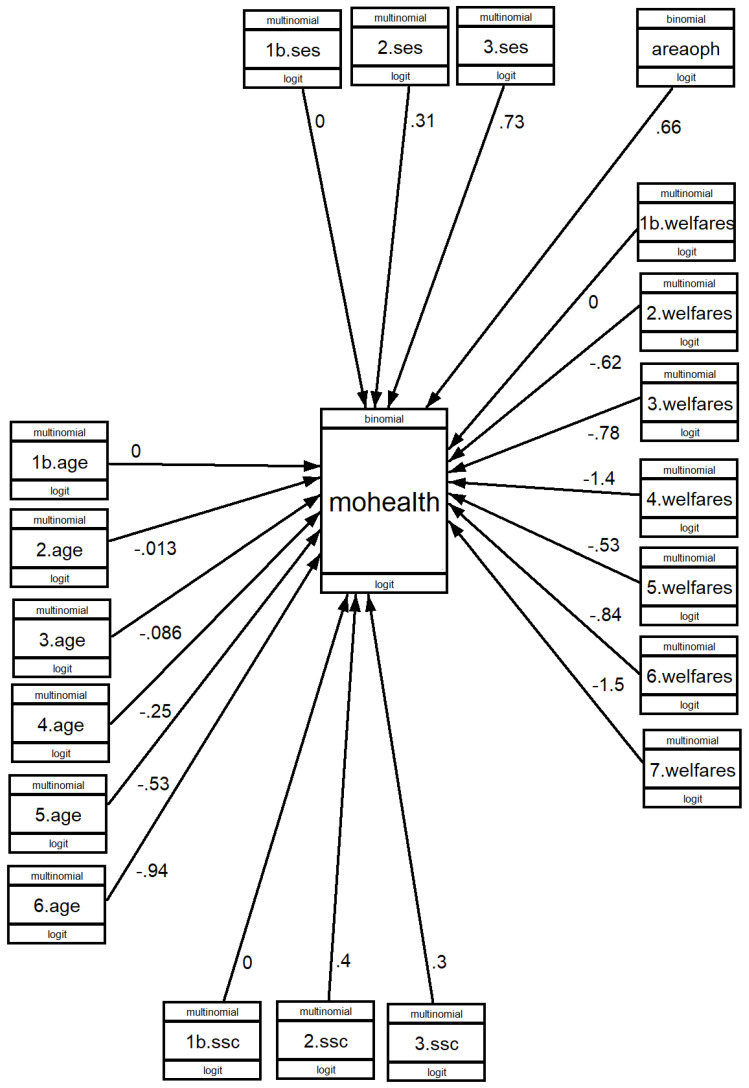
GSEM analysis of having health-based motivations to engage in LTPA according to social conditions. B values.

With the indicated models, the results are shown in Figure 2, Figure 3 and Figure 4, where the value of b can be seen. Table S8 shows the Odds Ratio values, which give the ratios and result from calculating the exponent of b (exp(b)), as well as the significance of the association (*p* > z).

#### 3.3.1. Engaging in LTPA

Age significantly impacts the practice of LTPA. While there are no significant differences between the 61–65 age group and the 66–70 age group, from the 71–75 age group onwards, there are very large differences. Gender also plays a role; for every 100 women practising sport and physical activities, 111 men do so. The higher the level of SES, the greater the practice of LTPA. The same occurs with SSC.

Habitat has a significant influence, but in comparison with the bivariate analysis, when analysed at the multivariate level, the extent of that influence varies, from small- and medium-sized cities compared to rural areas and towns. Controlling for the influence of the other variables, the weight of rural areas and towns relative to small- and medium-sized cities is 140 to 100. However, comparing large cities with small- and medium-sized cities, there is a significant decrease in large cities, with OR of 0.72. This tells us that for every 100 who practice physical and sport activities in big cities, 139 do so in small and medium-sized cities (1/0.72 = 1.39).

Living in an area that is perceived by respondents to have many opportunities for physical activity also has a strong influence, such that when many opportunities are perceived to exist, the probability is 175 compared to 100. The welfare state model of the country where people live is the most influential contextual factor. The difference between the countries of the Nordic model and the Mediterranean model is enormous, with the highest being 0.10. The differences between the Nordic model and the other models are also important.

#### 3.3.2. Engaging in LTPA That Meets the Minimum Time and Intensity Recommended by WHO

Age has a major impact on the performance of LTPA that meets the minimum recommended by WHO. Using the 61–65 age group as a base, the ratio for the 71–75 age group is 0.72 times lower, and for the 86 and over age group it is 0.18 times lower. SES has a very significant influence, so that for every 100 lower-class SES individuals, we have 145 middle-class and 213 upper-class SES individuals. SSC also has a significant influence even after controlling for the effect of SES, so that for every 100 people practising LTPA who consider themselves to be lower-class, there are 123 middle-class and 137 upper-class.

There are significant differences between those living in areas where there are many opportunities for PA and those living in areas where there are few opportunities for physical activity, with OR of 1.69. After controlling for other variables, those living in small- or medium-sized cities are more likely to engage in LTPA that meets the WHO minimum recommendations than those living in large cities but are less likely than those living in rural areas or villages. People living in countries of the Nordic welfare state model are the most likely to be physically active (LTPA) in ways that meet WHO recommendations.

#### 3.3.3. Engaging in LTPA for Health Reasons

There is no significant difference between people in the 61–65, 66–70 and 71–75 age groups in terms of health-driven motivation to engage in LTPA. The differences start to become significant in the 76–80 age group and increase in the groups of ages 81 and older.

SES is associated with health motivation to practice LTPA in a positive and highly significant way, with an OR of lower to middle SES of 1.37 and upper SES of 2.08. SSC is significantly associated if we compare lower class with middle class and upper class.

Living in areas where there are many opportunities for PA is significantly associated with the OR being 1.93 between those living in areas where there are no opportunities and those living in areas where there are many opportunities. The welfare state model of the country in which people live has a major influence. Using the countries of the Nordic model as a base, the OR of the countries bordering the Nordic model is 0.59; that of the countries of the Continental model is 0.54; that of the countries of the Anglo-Saxon model is 0.46; that of the countries of the Mediterranean model is 0.25; and the lowest is the countries bordering the Mediterranean model, at 0.21.

## 4. Discussion

To summarise the most relevant aspects of the results obtained on LTPA among older people in the EU, we can say that although 2/3 do engage in some LTPA, only 1/3 meet the minimum guidelines recommended by the WHO. Of those who engage in some kind of activity, 40.4% cite health motivations. The likelihood of engaging in LTPA varies according to social conditions, with relevant factors including socio-demographic factors (gender and age), but varies above all according to socio-economic factors, both at the individual (SES and SSC) and contextual level (habitat, infrastructure for physical activity in the area where people live, model of the country’s welfare state). The hypotheses are confirmed with regard to both individual and contextual factors.

Regarding the influence of age, the results of other studies are confirmed. As age increases, the percentage of people engaging in LTPA in general and, in particular, the number of those who meet the WHO time and intensity recommendations decreases, and it is from the age of 76 onward that this influence is most noticeable, when a sharp decline occurs. This confirms the results of other studies such as those of Mummery et al. [53] and Sims et al. [54], and it contradicts the results of authors who believe in a staggered decline, such as Ashe et al. [14], Frank et al. [51] and McGuire et al. [52]. This decline can also be perceived in health motivation.

Gender has a significant influence on LTPA and meeting WHO recommendations, with men’s activity being higher than that of women. This research confirms the results of Alvariñas et al. [58], but in a limited way, as gender does not influence health motivation within the multivariate analysis. While there was association at the bivariate level, it is no longer significant at the multivariate level. Upon introducing the SES, it takes over some of the weight that had corresponded to gender. It is not gender that has an influence, but rather socio-economic variables that are more detrimental to women than men, which in some ways contradicts the findings of other research that does not control the association between gender and physical activity with other socio-economic variables [71,72]. The explanation by Ball et al. [58] focuses on the influence SES exerts LTPA, such as negative PA experiences in early life for low SES strata, higher priority given to watching TV or work commitments at an early age, which help explain the reason for these differences. These factors are negative in low SES compared to upper SES. It is therefore essential to carry out multivariate association analyses, because if we only carry out bivariate analyses, the influence of other intervening variables cannot be observed.

Among the socio-economic factors, it is crucial to take individual and contextual factors into account. If we look at the influence of SES on LTPA, it is the most influential variable of all, with an OR of upper SES compared to low SES of 3.02. In the case of LTPA that meets WHO recommendations, it is also the highest OR, at 2.13. The same for the health-driven motivation for LTPA, which has an OR of 2.08.

This confirms the results of previous studies such as those of Kheifets [41], Chen et al. [38], Marqués [28], Azagba and Sharaf [16], Lobaszewski et al. [27], Borodulin et al. [42], Cerin and Leslie [22], Azevedo et al. [17] and Satariano et al. [32]. It is possible that in non-Western societies there may not be such a clear relationship or in studies that associate other types of PA in addition to LTPA, but in the case of LTPA among older people in European societies, the association is clear. This confirms the results that Stalsberg and Pedersen (2018) found after analysing the different studies carried out on this subject.

If we analyse the results of the association of LTPA, WHO-compliant LTPA and health-driven motivation to be physically active, with bivariate SES in each of the EU countries, nearly all of them show a positive and significant relationship (Table S9). The countries where the association is positive and significant (*p* < 0.05) for the three dependent variables are France, Germany-West, Italy, Denmark, Ireland, Germany-East, Finland, Austria, Czech Republic, Estonia, Hungary, Latvia, Lithuania, Slovakia, Slovenia and Bulgaria. The countries where the association is positive and significant only for the variables LTPA and engage in LTPA that meets the minimum WHO recommendations are the Netherlands, Northern Ireland, Spain and Cyprus (Republic). The only two countries where the association is not positive or not significant are Greece and Croatia.

Although most studies only consider target SES, our study confirms that it is necessary to include SSC as well, as even after controlling at the multivariate level for the effect of target SES, SSC still has a significant positive influence. In this respect, we consider to be relevant the indications of Singh-Manoux et al. [45], Adler et al. [46], Frerichs et al. [47] and Yang et al. [73]. Between two people belonging to the same SES as measured by objective indicators, if one of them subjectively considers themselves to be of higher social class than the other, the one who considers themselves to be of higher social class is more likely to engage in LTPA, to meet the WHO criteria and to have health-driven motivations for engaging in PA.

With regard to contextual factors, the influence of habitat confirms the results of other studies such as those of Muntner et al. [59], with people living in rural areas being the most active, as opposed to other studies that consider urban dwellers to be the most active [60]. Regarding the influence of the welfare state model on the practice of LTPA, the conclusions of Heinemann [62] are fulfilled, with the Nordic model states being those that engage in the most activity. The countries of the Mediterranean model are the least active, even less so than the surrounding countries of the former Soviet Union, highlighting the relevance of the conclusions of Fernández [67], which note the importance given to physical activity in these countries.

In terms of recommendations for future research, we would like to highlight the importance of carrying out qualitative studies to delve deeper into the reasons for not taking part in sport and physical activities, in an effort to make the appropriate recommendations and rectify the problem. In the present study, we analyse all LTPA together (whether or not they are sport-related), but we are aware that another specific study should be done to analyse the socio-economic differences between people who practise sport and those who do other types of physical activity during their leisure time. We cannot analyse this in the present article because it would be setting new objectives and analyses that would exceed the recommended length.

The Eurobarometer has some limitations. It clearly differentiates between what are sport-related physical activities (i.e., related to physical exercise such as swimming, training in a gym or sports club, running in the park, etc.) and what are other types of leisure-related physical activities (such as cycling, dancing, gardening, etc.), but it leaves out transport-related physical activities that are not performed for leisure, work and housework. We believe that future studies should differentiate between transport-related physical activity, occupational physical activity, housework-related physical activity and leisure time physical activity. Perhaps this could lead to more specific studies. We will write to the Eurobarometer to encourage the inclusion of questions on the different domains of PA in future surveys. Regarding the number of respondents, we believe that Eurobarometer should increase the number of surveys conducted in order to be able to carry out data analysis on specific groups. This would allow for drawing more definitive results.

## 5. Conclusions

In the countries of the European Union, there is a decline in the percentage of the population involved in LTPA that meets the guidelines of the WHO. Simultaneously, there has been an increase in the population over 65 years of age, from 17.6% in 2010 to 20.8% in 2021. This population is the most affected by inactivity. In the 61–65 age group, the percentage of people practising LTPA that meet WHO recommendations is 38.8%. However, from the age of 70 onwards, activity begins to decrease significantly. From the age of 76 onwards, this decrease is very high, with only 25.6% of the population in the 76–80 age group practising sport or physical activity that meets WHO recommendations.

There are differences in LTPA that meets WHO recommendations by the older population based on individual and contextual factors. The welfare state model of the countries where people live has a very significant influence. In the Nordic model countries, the average percentage is 51.4%, whereas in the Mediterranean model countries, it is only 14.4%. That is, in the Mediterranean model, activity is just over 1/4 that of the Nordic model. At a contextual level, the perception of opportunities to engage in LTPA in the area of residence is also very important.

SES also plays an important role, as 17.9% of those of low, 31.5% of middle and 47.3% of upper status engage in LTPA that meets the WHO minimum requirements. SSC is also important, having a significant influence even after removing the effect of SES.

Gender also plays a role, with men being more likely to engage in LTPA than women. Similarly, individual and contextual factors influence health-driven motivation for LTPA, with the exception that gender ceases to have a significant influence when associated at the multivariate level.

## Data Availability

All surveys of the European union are available in https://europa.eu/eurobarometer/surveys/browse/all/series/4961 (accessed on 4 October 2022).

## Supplementary Materials

The following supporting information can be downloaded at: https://www.mdpi.com/article/10.3390/healthcare11091247/s1, Table S1: Transformation of sport and physical activity variables; Table S2: Intensity of physical activity/sport according to WHO criteria applied to the Eurobarometer survey; Table S3: Independent variables and categories; Table S4: Percentage of population aged 65 and over in EU countries. Trend between 2010 and 2021; Table S5: Engagement in physical activity that meets WHO recommendations, according to age; Table S6: Bivariate results of physical-sports practice and health motivation; Table S7: Physical activity or sport practice and health motivation according to EU countries’ welfare state model (weighted percentage); Table S8: GSEM analysis of the variables engaging in sport or physical activity, meeting the minimum recommended by WHO, having health-driven motives, according to the social conditions. OR values and significance; Table S9. Impact of SES by country.
