# Supplementary material for: Ageing, Leisure Time Physical Activity and Health in Europe
Source: Healthcare (Basel). 2023 Apr 27;11(9):1247. doi: 10.3390/healthcare11091247 (PMC10178047; doi:10.3390/healthcare11091247)
Supplement: Supplementary file 1 [file healthcare-11-01247-s001.zip › healthcare-2263888-supplementary.pdf]

healthcare

MDPI

Supplementary materials

Table S1. Transformation of sport and other recreational physical activity variables in LTPA.

Wording of Questions	Original Categories	Final Wording	Final Categories
QB1 How often do you exercise or play sport?	5 times a week or more<br>3 to 4 times a week<br>1 to 2 times a week<br>1 to 3 times a month<br>Less often<br>Never<br>DK	Engages in leisure time physical activity (LTPA)	Yes
QB2 And how often do you engage in other physical activity such as cycling from one place to another, dancing, gardening, etc.? (recreational reasons)	5 times a week or more<br>3 to 4 times a week<br>1 to 2 times a week<br>1 to 3 times a month<br>Less often<br>Never<br>DK		No

Source: Own preparation.

Table S2. Intensity of LTPA according to WHO criteria applied to the Eurobarometer survey.

		Vigorous-Intensity Physical Activity (Minimum 75 Minutes)	
		No	***Yes***
Moderate-intensity physical activity (minimum 150 minutes)	*No*	Does not meet minimum	Meets vigorous-intensity physical activity
	Yes	Meets moderate-intensity physical activity or the sum of moderate and vigorous intensity	Meets vigorous- and moderate-intensity physical activity

Source: Own preparation.

Table S3. Independent variables and categories.

Variables	Categories
Age	61–65 years (base for multivariate analysis)
	66–70 years
	71–75 years
	76–80 years
	81–85 years
	86 years and older
Gender	Female (base for multivariate analysis)
	Male
Socioeconomic status (ses)	Lower (base for multivariate analysis)
	Middle
	Upper

Subjective social class (ssc)	Lower (base for multivariate analysis)
	Middle
	Upper
Type of community in which they live (habitat)	Small or medium-sized city (base for multivariate analysis)
	Rural area or village
	Big city
Opportunities for sport and physical activities in the area of residence (areaoph)	Disagree (base for multivariate analysis)
	Agree
Welfare state (welfares)	Nordic model (base for multivariate analysis)
	Continental model
	Anglo-Saxon model
	Mediterranean model
	Bordering Nordic model
	Bordering Continental model
	Bordering Mediterranean model

Source: Own preparation.

Table S4. Percentage of population aged 65 and over in EU countries. Trend between 2010 and 2021.

	2010	2011	2012	2013	2014	2015	2016	2017	2018	2019	2020	2021	2010/2021
European Union - 27	17.6	17.8	18.0	18.3	18.7	19.0	19.3	19.7	20.0	20.2	20.6	20.8	3.2
Finland	17.0	17.5	18.1	18.8	19.4	19.9	20.5	20.9	21.4	21.8	22.3	22.7	5.7
Poland	13.6	13.6	14.0	14.4	14.9	15.4	16.0	16.5	17.1	17.7	18.2	18.7	5.1
Czechia	15.3	15.6	16.2	16.8	17.4	17.8	18.3	18.8	19.2	19.6	19.9	20.2	4.9
Slovakia	12.4	12.6	12.8	13.1	13.5	14.0	14.4	15.0	15.5	16.0	16.6	17.1	4.7
Netherlands	15.3	15.6	16.2	16.8	17.3	17.8	18.2	18.5	18.9	19.2	19.5	19.8	4.5
Slovenia	16.5	16.5	16.8	17.1	17.5	17.9	18.4	18.9	19.4	19.8	20.2	20.7	4.2
France	16.6	16.7	17.1	17.6	18.0	18.4	18.9	19.3	19.7	20.0	20.4	20.7	4.1
Portugal	18.3	18.7	19.0	19.4	19.9	20.3	20.7	21.1	21.5	21.8	22.1	22.4	4.1
Malta	14.9	15.7	16.4	17.2	17.7	18.2	18.5	18.8	18.8	18.7	18.5	18.9	4.0
Cyprus	12.5	12.7	12.8	13.2	13.9	14.6	15.1	15.6	15.9	16.1	16.3	16.4	3.9
Denmark	16.3	16.8	17.3	17.8	18.2	18.6	18.8	19.1	19.3	19.6	19.9	20.1	3.8
Hungary	16.6	16.7	16.9	17.2	17.5	17.9	18.3	18.7	18.9	19.3	19.9	20.3	3.7
Ireland	11.2	11.5	11.9	12.2	12.6	12.9	13.2	13.5	13.8	14.1	14.4	14.8	3.6
Croatia	17.8	17.7	17.9	18.1	18.4	18.8	19.2	19.6	20.1	20.6	21.0	21.4	3.6
Bulgaria	18.2	18.5	18.8	19.2	19.6	20.0	20.4	20.7	21.0	21.3	21.6	21.7	3.5
Greece	19.0	19.3	19.7	20.1	20.5	20.9	21.3	21.5	21.8	22.0	22.3	22.5	3.5
Romania	16.1	16.1	16.1	16.3	16.5	17.0	17.4	17.8	18.2	18.5	18.9	19.3	3.2
Italy	20.4	20.5	20.8	21.2	21.4	21.7	22.0	22.3	22.6	22.9	23.2	23.5	3.1
Spain	16.8	17.1	17.4	17.7	18.1	18.5	18.7	19.0	19.2	19.4	19.6	19.8	3.0
Estonia	17.4	17.4	17.7	18.0	18.4	18.8	19.0	19.3	19.6	19.8	20.0	20.3	2.9
Latvia	18.1	18.4	18.6	18.8	19.1	19.4	19.6	19.9	20.1	20.3	20.5	20.8	2.7
Lithuania	17.3	17.9	18.1	18.2	18.4	18.7	19.0	19.3	19.6	19.8	19.9	19.9	2.6
Belgium	17.2	17.1	17.4	17.6	17.8	18.1	18.2	18.5	18.7	18.9	19.1	19.3	2.1
Sweden	18.1	18.5	18.8	19.1	19.4	19.6	19.8	19.8	19.8	19.9	20.0	20.1	2.0

Austria	17.6	17.6	17.8	18.1	18.3	18.5	18.4	18.5	18.7	18.8	19.0	19.2	1.6
Germany	20.7	20.7	20.7	20.8	20.9	21.0	21.1	21.2	21.4	21.5	21.8	22.0	1.3
Luxembourg	14.0	13.9	14.0	14.0	14.1	14.2	14.2	14.2	14.3	14.4	14.5	14.6	0.6

Source: Own preparation based on Eurostat data.

Table S5. Engagement in LTPA that meets WHO recommendations, according to age.

	61–65 Years	66–70 Years	71–75 Years	76–80 Years	81–85 Years	86 Years and Older	Total
Moderate physical activity 150'	11.9	11.1	11.3	10.4	8.7	4.3	10.8
Vigorous physical activity 75'	2.4	2.1	2.1	1.8	1.5	0.5	2
Meets both guidelines	24.5	23.2	18.4	13.3	10.4	4.9	19.4
Meets any of the guidelines (sum of the three above)	38.8	36.4	31.8	25.6	20.6	9.8	32.3
Does not engage in LTPA, or engages but does not meet any WHO guidelines	61.2	63.6	68.2	74.4	79.4	90.2	67.7

Source: Own preparation based on Eurobarometer 88.4.

Table S6. Bivariate results of LTPA and health motivation.

		Engage in LTPA That Meets the Minimum WHO Recommendations (Ltpawho)	Sig. Level	Engage in LTPA Daily, Often or Sometimes (ltpa)	Sig. Level	Health-Driven Motivation to Engage in LTPA Among the Whole Population (mohealth)	Sig. Level
Age	61–65 years	38.8	***	71.2	***	43.9	***
	66–70 years	36.4		70.0		43.1	
	71–75 years	31.8		66.0		42.1	
	76–80 years	25.6		59.5		36.9	
	81–85 years	20.6		50.2		29.6	
	86 years and older	9.8		40.2		22.8	
Gender	Female	28.7	***	62.4	***	39.3	*
	Male	36.6		68.8		41.8	
Socioeconomic status	Lower	17.9	***	44.9	***	24.9	***
	Middle	31.5		65.4		39.1	
	Upper	47.3		84.9		58.2	
Subjective social class	Lower	25.0	***	54.4	***	30.5	***
	Middle	37.5		74.3		48.9	
	Upper	49.4		84.2		56.4	
Type of community in which they live	Small or medium-sized city	33.4	***	67.5	***	42.1	**
	Rural area or village	34.4		66.2		38.5	
	Big city	27.8		60.3		39.9	

Opportunities for sport and physical activities in the area of residence	Disagree	20.1	***	48.7	***	24.1	***
	Agree	37.4		72.3		47.3	
Welfare state	Nordic model	51.4	***	91.3	***	65.0	***
	Continental model	42.2		79.3		49.4	
	Anglo-Saxon model	27.3		64.6		37.4	
	Mediterranean model	14.4		37.3		21.3	
	Bordering Nordic model	31.7		65.1		44.0	
	Bordering Continental model	27.7		61.3		36.8	
	Bordering Mediterranean model	24.4		49.3		19.9	

Source: Own preparation based on Eurobarometer 88.4. Significance of the association: *, **, ***.

Table S7. LTPA and health motivation according to EU countries' welfare state model (weighted percentage).

	Engage in LTPA That Meets the Minimum WHO Recommendations (Ltpawho)	Engage in LTPA Daily, Often or Sometimes (ltpa)	Health-Driven Motivation to Engage in LTPA Among the Whole Population (mohealth)
1 Nordic model	51.4	91.3	65.0
SE - Sweden	49.3	92.7	73.6
FI - Finland	54.7	89.7	55.5
DK - Denmark	43.6	88.9	68.2
2 Continental model	42.2	79.3	49.4
NL - The Netherlands	60.3	92.7	60.2
LU - Luxembourg	46.8	86.0	51.8
DE-W - Germany - West	51.1	74.0	44.3
DE-E Germany East	47.8	78.5	50.5
FR - France	26.9	67.6	35.7
BE - Belgium	29.2	74.3	35.0
AT - Austria	26.3	63.2	34.3
3 Anglo-Saxon model	27.3	64.6	37.4
GB-GBN - Great Britain	27.1	64.9	34.0
IE - Ireland	26.3	64.5	40.1
GB-NIR Northern Ireland	26.7	63.7	42.5
4 Mediterranean model	14.4	37.3	21.3
ES -Spain	22.9	46.3	29.9
CY - Cyprus (Republic)	20.8	46.9	31.4

GR - Greece	16.1	44.4	25.8
MT - Malta	7.4	32.1	16.2
PT - Portugal	8.7	25.2	12.9
IT - Italy	7.9	30.7	12.9
5 Bordering Nordic model	31.7	65.1	44.0
EE - Estonia	36.3	64.3	45.3
LV - Latvia	33.8	70.5	50.9
LT - Lithuania	25.7	61.7	37.4
6 Bordering Continental model	27.7	61.3	36.8
SI - Slovenia	31.3	78.8	63.1
SK - Slovakia	30.7	63.6	30.1
HU - Hungary	34.8	55.9	29.9
CZ - Czech Republic	17.7	58.9	31.3
PL - Poland	22.4	46.0	24.7
7 Bordering Mediterranean model	24.4	49.3	19.9
HR - Croatia	27.5	56.6	27.7
BG - Bulgaria	20.6	45.6	14.3
RO - Romania	22.7	45.6	17.8
Total EU	32.3	65.3	40.4

Source: Own preparation based on Eurobarometer 88.4.

Table S8. GSEM analysis of the variables engaging in LTPA, meeting the minimum recommended by WHO, having health-driven motives, according to the social conditions. OR values and significance.

	**ltpa**	**Sig. Level**	**ltpawho**	**Sig. Level**	**mohealth**	**Sig. Level**
	exp(b) (OR)	*p* > z	exp(b) (OR)	*p* > z	exp(b) (OR)	*p* > z
Age						
61–65 years	1.00		1.00		1.00	
66–70 years	0.97	0.666	0.91	0.140	0.99	0.831
71–75 years	0.78	0.000	0.72	0.000	0.92	0.175
76–80 years	0.61	0.000	0.56	0.000	0.78	0.001
81–85 years	0.42	0.000	0.45	0.000	0.59	0.000
86 years and older	0.25	0.000	0.18	0.000	0.39	0.000
ses: Socioeconomic status						
Basic or less	1.00		1.00		1.00	
Secondary	1.50	0.000	1.45	0.000	1.37	0.000
Higher	3.02	0.000	2.13	0.000	2.08	0.000
ssc: Subjective social class						
Low	1.00		1.00		1.00	
Middle	1.50	0.000	1.23	0.000	1.49	0.000
Upper	1.50	0.001	1.37	0.001	1.34	0.001
areaoph: The area where they live offers many opportunities for physical activity						
Disagree/Agree	1.75	0.000	1.69	0.000	1.93	0.000
Habitat						
Rural area or village	1.40	0.000	1.36	0.000		
Small or medium-sized city	1.00		1.00			
Big city	0.72	0.000	0.78	0.000		
welfares: Welfare state model of the country where they live						
Nordic model	1.00		1.00		1.00	

Continental model	0.38	0.000	0.70	0.000	0.54	0.000
Anglo-Saxon model	0.27	0.000	0.49	0.000	0.46	0.000
Mediterranean model	0.10	0.000	0.25	0.000	0.25	0.000
Bordering Nordic model	0.26	0.000	0.61	0.000	0.59	0.000
Bordering Continental model	0.21	0.000	0.46	0.000	0.43	0.000
Bordering Mediterranean model	0.14	0.000	0.44	0.000	0.21	0.000
		Gender				
Female/Male	1.11	0.035	1.27	0.000		

Source: Own preparation based on Eurobarometer 88.4.

Table S9. Impact of SES by country.

			Engage in LTPA That Meets the Minimum WHO Recommendations (Ltpawho)	Sig. Level	LTPA	Sig. Level	Health-Driven Motivation to Engage in LPTA, Among the Whole Population	Sig. Level	Association + (Positive), - (Negative) and 0 (Lack of Linear Significance)
FR - France	SES	Lower	10.1%	***	48.5%	***	23.2%	**	+, +, +
		Middle	25.3%		69.6%		38.6%		
		Upper	40.7%		81.4%		42.5%		
BE - Belgium	SES	Lower	23.4%		60.9%	**	20.3%	*	+, +, +
		Middle	28.8%		74.7%		39.7%		
		Upper	34.4%		82.8%		37.6%		
NL - The Netherlands	SES	Lower	36.1%	***	80.6%	***	44.4%		+, +, +
		Middle	52.2%		89.0%		61.5%		
		Upper	72.5%		97.2%		61.5%		
DE-W - Germany - West	SES	Lower	32.4%	***	52.8%	***	25.0%	***	+, +, +
		Middle	45.5%		73.9%		44.1%		
		Upper	67.4%		91.1%		60.0%		
IT - Italy	SES	Lower	4.5%	***	20.9%	***	9.0%	**	+, +, +
		Middle	10.2%		29.5%		11.4%		
		Upper	26.2%		64.3%		28.6%		
LU - Luxembourg	SES	Lower	37.0%		63.0%	***	29.6%	*	+, +, (+)
		Middle	46.3%		85.1%		58.2%		
		Upper	48.6%		95.7%		54.3%		
DK - Denmark	SES	Lower	25.0%	***	71.9%	***	53.1%	***	+, +, +
		Middle	34.6%		83.1%		60.8%		
		Upper	48.6%		94.7%		74.3%		
IE - Ireland	SES	Lower	10.9%	***	50.0%	***	28.3%	***	+, +, +
		Middle	29.5%		63.8%		36.2%		
		Upper	50.8%		86.2%		63.1%		

GB-GBN - Great Britain	SES	Lower	20.3%		49.7%	***	25.9%	*	+, +, +
		Middle	29.4%		69.9%		36.2%		
		Upper	32.9%		82.9%		44.7%		
GB-NIR Northern Ireland	SES	Lower	10.3%	*	48.7%	*	33.3%		+, +, +
		Middle	35.0%		66.7%		43.3%		
		Upper	42.9%		92.9%		64.3%		
GR – Greece	SES	Lower	17.7%		44.3%		22.2%		0, 0, +
		Middle	15.6%		45.9%		28.4%		
		Upper	18.2%		41.8%		30.9%		
ES -Spain	SES	Lower	19.9%	*	40.7%	**	27.0%		+, +, +
		Middle	32.5%		57.8%		34.9%		
		Upper	36.4%		81.8%		54.5%		
PT - Portugal	SES	Lower	8.2%		23.0%		10.9%	*	0, +, +
		Middle	11.1%		31.9%		16.7%		
		Upper	8.3%		33.3%		33.3%		
DE-E Germany East	SES	Lower	22.0%	***	61.0%	***	31.7%	**	+, +, +
		Middle	45.1%		77.0%		49.6%		
		Upper	61.7%		93.3%		65.0%		
FI - Finland	SES	Lower	35.4%	***	76.8%	***	39.0%	**	+, +, +
		Middle	50.9%		88.9%		56.2%		
		Upper	63.2%		95.7%		61.2%		
SE - Sweden	SES	Lower	48.0%		76.0%	***	56.0%	*	0, +, +
		Middle	46.9%		88.5%		66.9%		
		Upper	50.5%		95.0%		76.7%		
AT - Austria	SES	Lower	10.2%	***	42.4%	***	22.0%	**	+, +, +
		Middle	24.8%		61.5%		31.2%		
		Upper	48.6%		82.4%		48.6%		
CY - Cyprus (Republic)	SES	Lower	11.2%	*	31.5%	***	23.6%		(+), (+), +
		Middle	28.4%		63.0%		37.0%		
		Upper	20.8%		50.0%		41.7%		
CZ - Czech Republic	SES	Lower	3.3%	**	26.7%	***	10.0%	**	+, +, +
		Middle	16.0%		59.9%		31.0%		
		Upper	31.0%		72.4%		43.1%		
EE - Estonia	SES	Lower	25.7%	*	54.1%	**	29.7%	***	+, +, +
		Middle	32.9%		59.9%		42.2%		
		Upper	45.2%		75.0%		56.5%		
HU - Hungary	SES	Lower	27.2%	***	44.9%	***	23.8%	*	+, +, +
		Middle	30.9%		57.0%		30.3%		
		Upper	61.3%		79.0%		43.5%		
LV - Latvia	SES	Lower	19.6%	*	54.9%	**	33.3%	**	+, +, +
		Middle	37.5%		70.5%		49.5%		
		Upper	40.0%		80.0%		64.7%		
LT - Lithuania	SES	Lower	14.5%	*	47.0%	***	24.8%	***	+, +, +
		Middle	26.7%		61.9%		36.2%		
		Upper	29.1%		76.1%		52.1%		
MT - Malta	SES	Lower	2.3%	*	27.7%		15.4%		(+), (+), (+)
		Middle	11.9%		39.3%		14.3%		
		Upper	10.0%		30.0%		30.0%		
PL - Poland	SES	Lower	15.2%		39.4%	*	17.2%	**	+, +, +

		Middle	22.4%		44.9%		23.1%		
		Upper	29.6%		61.1%		42.6%		
SK - Slovakia	SES	Lower	9.4%	***	51.6%	*	31.3%	*	+, +, (+)
		Middle	34.4%		64.7%		26.5%		
		Upper	45.0%		77.5%		47.5%		
SI - Slovenia	SES	Lower	26.5%		60.2%	***	49.4%	**	+, +, +
		Middle	29.1%		79.1%		63.8%		
		Upper	37.8%		93.9%		73.5%		
BG - Bulgaria	SES	Lower	12.5%	*	43.8%	*	6.3%	**	+, (+), +
		Middle	17.6%		40.1%		10.6%		
		Upper	33.9%		59.7%		29.0%		
RO - Romania	SES	Lower	22.7%		38.1%	*	9.3%	**	(+), +, +
		Middle	21.2%		44.4%		18.2%		
		Upper	35.6%		64.4%		35.6%		
HR - Croatia	SES	Lower	30.5%		58.5%		28.0%		-, -, 0
		Middle	30.8%		56.2%		26.9%		
		Upper	10.0%		53.3%		30.0%		
Total	SES	Lower	17.9%	***	44.9%	***	24.1%	***	
		Middle	31.5%		65.4%		39.1%		
		Upper	47.3%		84.9%		58.2%		
		Total	32.3%		65.3%		40.4%		

+, +, +: Positive significative progressive association in all three variables (LTPA; Engage in physical activity that meets the minimum WHO recommendations; Health-driven motivation to engage in physical activity, among the whole population). (+): The association is positive, but it is not progressive. -: Negative association. 0: No association. Significance of the association: *, **, ***.
